# Treatment resistance in psychiatry: state of the art and new directions

**DOI:** 10.1038/s41380-021-01200-3

**Published:** 2021-07-13

**Authors:** Oliver D. Howes, Michael E. Thase, Toby Pillinger

**Affiliations:** 1 Institute of Psychiatry, Psychology and Neuroscience, King’s College London, London, UK; 2 MRC London Institute of Medical Sciences, Faculty of Medicine, Imperial College London, Hammersmith Hospital Campus, London, UK; 3 H Lundbeck A/s, St Albans, UK; 4 Department of Psychiatry, Perelman School of Medicine, University of Pennsylvania and Corporal Michael J. Crescenz VA Medical Center, Philadelphia, USA

**Keywords:** Psychiatric disorders, Diagnostic markers

## Abstract

Treatment resistance affects 20–60% of patients with psychiatric disorders; and is associated with increased healthcare burden and costs up to ten-fold higher relative to patients in general. Whilst there has been a recent increase in the proportion of psychiatric research focussing on treatment resistance (*R*^2^ = 0.71, *p* < 0.0001), in absolute terms this is less than 1% of the total output and grossly out of proportion to its prevalence and impact. Here, we provide an overview of treatment resistance, considering its conceptualisation, assessment, epidemiology, impact, and common neurobiological models. We also review new treatments in development and future directions. We identify 23 consensus guidelines on its definition, covering schizophrenia, major depressive disorder, bipolar affective disorder, and obsessive compulsive disorder (OCD). This shows three core components to its definition, but also identifies heterogeneity and lack of criteria for a number of disorders, including panic disorder, post-traumatic stress disorder, and substance dependence. We provide a reporting check-list to aid comparisons across studies. We consider the concept of pseudo-resistance, linked to poor adherence or other factors, and provide an algorithm for the clinical assessment of treatment resistance. We identify nine drugs and a number of non-pharmacological approaches being developed for treatment resistance across schizophrenia, major depressive disorder, bipolar affective disorder, and OCD. Key outstanding issues for treatment resistance include heterogeneity and absence of consensus criteria, poor understanding of neurobiology, under-investment, and lack of treatments. We make recommendations to address these issues, including harmonisation of definitions, and research into the mechanisms and novel interventions to enable targeted and personalised therapeutic approaches.

## Introduction

**Fig. 1:**
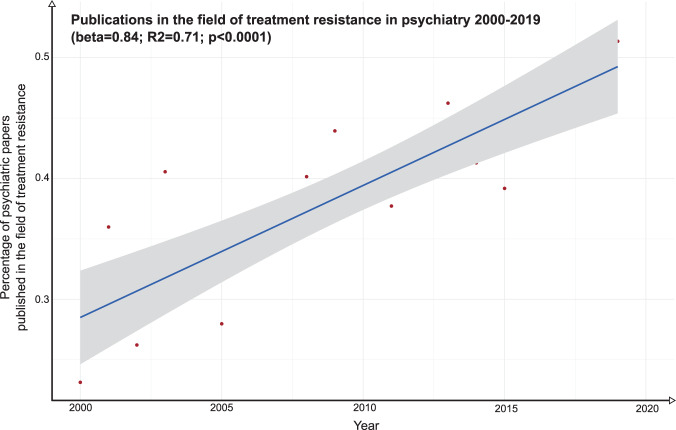
Scatterplot showing the relationship between the number of papers published in the field of treatment resistance in psychiatry and time (2000–2019). The number of papers published on treatment resistance in psychiatry are presented as a percentage of the total number of publications in the field of psychiatry overall. The solid blue line corresponds to the regression estimate with the corresponding 95% confidence interval, indicated by grey shading, showing a significant increase in the percentage of psychiatric research focusing on treatment resistance over the last two decades.

The discovery of medications with clinically meaningful antidepressant and antipsychotic effects in the mid-twentieth century was a landmark in the treatment of mental disorders. However, soon afterwards it was recognised that in some patients, their condition showed limited or no response to these drugs [1–3]. Where an illness does not respond despite an adequate course of treatment, it is generally termed treatment resistant. Treatment resistance is now recognised across a range of psychiatric disorders, including schizophrenia, major depressive disorder (MDD), bipolar affective disorder [4], and obsessive compulsive disorder (OCD) [5]. Despite this, treatment resistance was not a focus of psychiatric drug development for decades and, to date, only one treatment, clozapine, is a licensed monotherapy for treatment resistance in psychiatry, and then specifically for schizophrenia. However, there are signs of change, with companies developing drugs for treatment resistance for a number of psychiatric disorders [6–8], and there is now broad recognition that a ‘one size fits all’ approach has reached the limits of effectiveness [9]. To evaluate research interest in treatment resistance in psychiatry, we conducted an analysis of scientific publications on treatment resistance in psychiatry between 2000–2019 inclusive (see eAppendix 1 for methods and full results). This shows a significant year-on-year increase in the number of papers published focussing on treatment resistance in psychiatry, after accounting for the increase in total number of publications over time (*β* = 0.84, *R*^2^ = 0.71, *p* < 0.0001, Fig. 1). It is thus timely to review the concepts of treatment resistance used in psychiatry, its prevalence and burden across disorders, and to consider current and future therapeutic directions.

### The origins of the concept of treatment resistance

The use of the term treatment resistance in psychiatry pre-dates the discovery of antipsychotics, antidepressants and mood stabilisers. More than a century ago, Freud used the term ‘resistance’ to describe unconscious mental reactions and behaviour exhibited during psychoanalysis that inhibited the response to therapy; identifying and addressing resistance is, in this context, considered therapeutic [10]. The term was also used in the 1930s when insulin coma therapy was used for schizophrenia, to describe the scenario where patients maintained consciousness despite delivery of large insulin doses [11]. Early reports of treatment resistance to antipsychotics and antidepressants did not include the Freudian concept of an unconscious mental reaction, but focused instead on inadequate symptomatic response to treatment [1–3]. This concept was crystallised in the late 1980s [12] with the demonstration of clozapine’s efficacy over chlorpromazine in patients with schizophrenia whose illness had not responded to at least three previous antipsychotic trials. Since then, a number of criteria have been developed to define treatment resistance in different disorders.

### Current definitions of treatment resistance across disorders

**Fig. 2:**
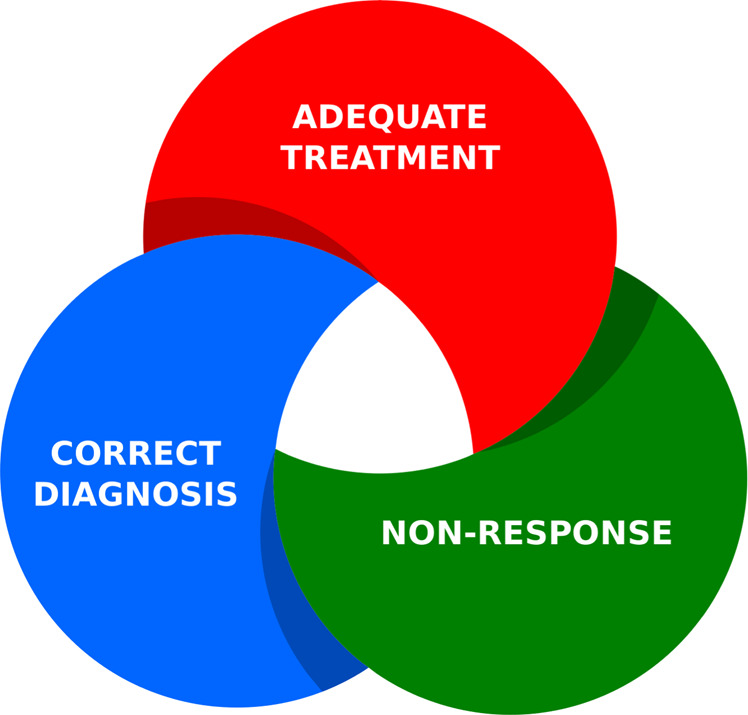
Treatment resistance consists of three core components. Establishing treatment resistance requires concurrent confirmation of the following: 1) that the correct psychiatric diagnosis has been made; 2) that a patient has received adequate treatment; 3) that symptoms have not adequately responded despite treatment.

We conducted a systematic review of national and international consensus definitions of treatment resistance for common psychiatric diagnoses (see eAppendix 2 for full search strategy). This identified 23 guidelines: 9 for schizophrenia; 10 for depression; 1 for the depressed phase of bipolar affective disorder; and 3 for OCD. The criteria used in these guidelines are summarised in Table 1. These highlight three core components required to establish treatment resistance seen across disorders and guidelines; these are that the correct diagnosis has been made, that adequate treatment has been given, and that there has been inadequate response (see Fig. 2).

**Table 1:** Definitions of treatment resistance used across psychiatry from consensus statements and national/international guidelines.

Guideline	Diagnosis Specified?	Diagnostic criteria specified?	Minimum number of treatment trials (drug trials unless specified)	Treatment duration (per drug)	Dose thresholds		Assessment of adherence	Criteria for inadequate response
*Schizophrenia*								
APA [95]	Diagnosis of schizophrenia required	Diagnosis should use DSM-IV-R criteria	Two antipsychotics at least one of which is a second-generation antipsychotic	≥6 weeks		Therapeutic range	‘Assess medication adherence may be assessed by patient/caregiver report, pill counts, prescription refill counts, and antipsychotic blood levels.’	‘A clinically inadequate response…persistent suicidal ideation or behaviour that has not responded to other treatments’
BAP [96]	Diagnosis of schizophrenia required	Diagnosis should use DSM/ICD criteria	Two, one of which should be an antipsychotic with an established favourable efficacy profile in comparison with other antipsychotics	‘Adequate’		‘Adequate’	’Poor adherence and substance use should be excluded as causes of the poor response to antipsychotic’	‘Schizophrenic illness that has proved unresponsive to adequate trials of two or more antipsychotics’
IPAP [97]	Diagnosis of schizophrenia required	Criteria not specified	Two antipsychotics	4–6 weeks		‘Adequate’	Not specified	‘Psychosis… after adjusting dose’
Maudsley [98]	Diagnosis of schizophrenia implied	Criteria not specified	Two antipsychotics	2–3 weeks for first trial in first-episode; 6-weeks for subsequent trials		At least minimum effective dose, then titrate to response	Not specified	Not specified
MOHS [99]	Diagnosis of schizophrenia implied	Criteria not specified	Two antipsychotics	‘Adequate’		‘Adequate’	Not specified	‘Illness has not responded adequately to treatment’
NICE [100]	Diagnosis of schizophrenia implied	Criteria not specified	Two antipsychotics, one of which should be a non-clozapine second-generation antipsychotic	Not specified		‘Adequate’	‘Establish that there has been adherence to antipsychotic medication’	’Illness has not responded adequately to treatment’
RANZCP [101]	Diagnosis of schizophrenia required	Diagnosis should use DSM/ICD criteria	Two antipsychotics Recommends both first and second trial to be an atypical antipsychotic	6–8 weeks		Specific dosages specified	Not specified	‘Poor response’
TRRIP [22] (consensus definition)	Diagnosis of schizophrenia required	Diagnosis should be ‘based on validated criteria’	Two different antipsychotics	≥6 weeks		Minimum dose equivalent 600 mg chlorpromazine per day	‘≥80% of prescribed doses taken. Assess adherence using at least 2 sources. Antipsychotic plasma levels monitored at least once.’	‘Interview using standardised rating scale (e.g., PANSS), ongoing symptoms of at least moderate severity, and at least moderate functional impairment measured using a validated scale’
WFSBP [102]	Diagnosis of schizophrenia required	Diagnosis should use DSM/ICD criteria	Two antipsychotics ‘One of which should be an atypical antipsychotic’	6–8 weeks		‘Recommended dosage’	Adherence should be ensured, if necessary, by checking drug concentrations	No improvement at all or only insufficient Improvement
*Depression*								
APA [103, 104]	Diagnosis of depression required	Diagnosis should use DSM-IV	Not defined numerically, any class (or same class)	≥8 weeks. Review dose at 4–8 weeks, consider dose increase		‘Upper limit of a medication dose’	‘Assess…treatment adherence’	‘Minimal or no improvement in symptoms’
BAP [105]	Diagnosis of depression required	Diagnosis should use DSM/ICD	Not defined numerically, any class (or same class)	≥4 weeks		‘Recommended therapeutic dose’	‘Check the adequacy of treatment including dose and non-adherence’	‘Inadequate response’
CANMAT [106, 107]	Diagnosis of depression required	Diagnosis should use DSM	Two antidepressants, classes not defined	Not defined		Not defined	Not defined	Not defined
GSRD [108]	Diagnosis of depression required	Diagnosis should use DSM-IV	Two antidepressants, any class (or same class)	≥4 weeks		‘Optimal dose of prescribed antidepressant (at least as high as the lowest dose defined effective in product data sheet)’	Not defined	Persistent HAM-D-17 [109] score ≥ 17
Maudsley [98]	Diagnosis of depression is implied	Not specified	Three antidepressants (classes not specified)	≥3 weeks		At least minimum effective dose, titrate to response	Not defined	‘No effect’
McAllister-Williams et al. [110] (consensus definition)	Diagnosis of depression required	Criteria not specified	≥3 (multi-therapy-resistant depression) ‘it is recommended that the trials should not all be from the same class of drugs ‘	8 weeks		Maximum licensed or maximum tolerated (where maximum tolerated dose is minimal therapeutic dose)	‘The clinician is confident (based on clinical judgement and patient history) that the patient has been adherent’	Failure to ‘experience full or sustained remission of symptoms’
MOHS [111]	Diagnosis of depression required	Criteria not specified	Not defined numerically, any class (or same class)	≥4 weeks		‘Titrate…to the full therapeutic dose’	Not defined	‘Not responded to adequate trials’
NICE [112]	Diagnosis of depression required	Diagnosis should use DSM	Not defined numerically Initially treat with SSRI. If switching, consider alternative SSRI as first-line change	3-4 weeks		‘Therapeutic dose’	‘Check adherence’	‘Inadequate response’
RANZCP [113]	Diagnosis of depression required	Diagnosis should use DSM	Two antidepressants (classes not specified)	≥3 weeks		‘At the recommended therapeutic dose’	‘Ensure that the patient has been taking their medication as prescribed’	‘Lack of improvement’
WFSBP [114]	Diagnosis of depression required	Criteria not specified	Two antidepressants, any class (or same class)	‘Adequate’		‘Adequate’	‘Assessing adherence to the current treatment regimen is recommended’	‘Inadequate response’
*Bipolar affective disorder (depression)*								
Hidalgo-Mazzei et al. [115] (consensus definition)	Diagnosis of bipolar 1 or 2 disorder required	Diagnosis should use DSM criteria	2 (antipsychotic / mood-stabiliser)	8		Adequate therapeutic doses	‘Include continuous and rigorous medication adherence’	‘Failure to reach sustained remission’
*Obsessive compulsive disorder (OCD)*								
AACAP [116]	Diagnosis of OCD required	Criteria not specified	Two drug trials: either 2 trials of SSRI, or 1 trial of SSRI and 1 trial of clomipramine 1 trial of CBT	Drug: 10 weeks CBT: 8–10 sessions, or 6–8 sessions of exposure and response prevention		Maximum recommended or maximum tolerated doses	Not defined	‘Persistent and substantial OCD symptomatology’
International Treatment Refractory OCD Consortium [117] (consensus definition)	Diagnosis of OCD required	Criteria not specified	Non-response defined on scale of 1–10 depending on intensity of treatment, ranging from SSRI/CBT mono-therapy up to polytherapy +/− neurosurgery	Drug: 12 weeks CBT: not defined		‘At least moderate doses’	Not defined	‘Non-response’ defined as <25% Y-BOCS reduction and CGI 4, ‘refractory’ defined as ‘no change/ worsening with therapies’
Mataix-Cols et al. [118] (consensus definition)	Diagnosis of OCD required	‘Structured diagnostic interview’ as part of assessing diagnostic criteria for OCD	Not defined	Not defined		Not defined	Not defined	Non-response <35% reduction in the Yale-Brown Obsessive Compulsive Scale (Y-BOCS) plus clinical global impression scale improvement rated ≥3

For each definition of treatment resistance, the table summarises the requisite number of pharmacological treatment trials, the necessary treatment duration and dose, and how treatment adherence and response are assessed.

*APA* American Psychiatric Association, *BAP* British Association for Psychopharmacology, *IPAP* International Psychopharmacology Algorithm Project, *MOHS* Ministry of Health Singapore, *NICE* National Institute for Health and care Excellence, *RANZCP* Royal Australian and New Zealand College of Psychiatrists, *WFSBP* World Federation of Societies of Biological Psychiatry, *GSRD* European Group for the Study of Resistant Depression, *TRRIP* Treatment Response and Resistance in Psychosis (TRRIP) working group, *AACAP* American Academy of Child and Adolescent Psychiatry, *CANMAT* Canadian Network for Mood and Anxiety Treatments, *OCD* obsessive compulsive disorder.

However, whilst these core components are seen in the definitions of treatment resistance across disorders, there is considerable variation in the specific criteria used between different disorders. Surprisingly, marked variation in criteria is also seen within the same psychiatric diagnoses, particularly regarding how adequate pharmacotherapy is defined and how response to treatment is assessed. For example, the number of treatment trials that an individual should receive prior to a diagnosis of treatment resistance is, for some criteria for OCD and depression, either not defined, or inconsistent, ranging between 2–3 different drugs for depression. Furthermore, certain criteria for schizophrenia, depression, and OCD recommend using different drug classes (e.g., ensuring a trial of first and second-generation antipsychotics in some criteria for schizophrenia), while others do not make this specification. Moreover, the target drug dose is often not defined, despite some conditions having well-described dose-equivalents at which clinical response is expected [13]. Thus, subtherapeutic trials of treatments may be erroneously considered adequate trials, leading to an over-inclusive classification of treatment resistance. For OCD, definitions of treatment trials also are inconsistent on whether or not psychological therapy should be included as a treatment alongside pharmacological therapy. For some criteria, recommended treatment durations are either not defined, or range between 2–12 weeks. Treatments such as those delivered in long-acting injectable formulation may take months to reach steady state, thus there is the risk that shorter trials of certain medications may be inadequate [14, 15]. There is also a lack of clarity regarding use of some neuro-stimulatory treatments. For example, although most MDD guidelines discuss use of transcranial magnetic stimulation as a therapeutic option, advice regarding its role in the context of treatment resistance, whether it should be used as monojunctive or adjunctive therapy, and what position it should take in a treatment algorithm for resistant symptoms is often not stated.

Inherent to the concept of treatment resistance is the idea that there is inadequate treatment response. Of the 23 guidelines identified, we observed that criteria for defining inadequate treatment response varied widely and were not provided in two definitions. Three defined inadequate treatment response in absolute terms using statements such as ‘no effect’, four recommended assessments of quantitative change in symptom severity using symptom rating scales and thresholds to define response, and fourteen used amorphous terms such as ‘inadequate’ or ‘minimal’ response. Failure to objectively quantify symptom severity increases risk of inter-rater variability, where one clinician may differ from another based on their subjective opinions of what constitutes treatment response. Using ‘no effect’ as a criterion is likely to be too restrictive in most clinical scenarios given natural variation in the course of disorder and placebo response [16], even without considering what level of therapeutic response might be adequate. In contrast, many criteria focus on definitions of failure to meet a minimum response threshold, which allows for natural variation and placebo response, but implies there may be a response, albeit inadequate. Failure to achieve a specific therapeutic response, but achieving a partial response to treatment, is not equivalent to no change in symptoms at all, particularly where the response threshold is high, and results in different conceptualisations of treatment resistance and potentially leading to very different patients being included in studies. Although defining response in a binary fashion is necessary to guide subsequent management (e.g., a change in medication), grouping patients together with putatively different presentations (i.e., absolute non-responders with partial-responders) may result in ineffective and inappropriate treatment being offered to certain patients. Partial response to a given pharmacological treatment could indicate that the drug is having some benefit, but the dose or duration of treatment may not be sufficient. In contrast, absolute non-response to treatment suggests the drug’s mechanism of action is not relevant for this patient, and thus a treatment with a medication with an alternate mode of action may be more appropriate. The potential consequence of grouping partial-responders and absolute non-responders together is that absolute non-responders may then be offered a treatment that is ineffective, and partial-responders may be unnecessarily offered riskier or experimental treatments. Inconsistency and lack of clarity in definitions of treatment resistance also has implications for interpretation of findings across clinical trials, as characteristics of patients in one study population may contrast markedly with the characteristics in another. Inconsistencies in definitions of treatment resistance between studies may be responsible for surprising results from meta-analyses examining efficacy of pharmacological treatments in treatment resistant psychiatric disorders [17, 18].

We were unable to identify operationalised definitions of treatment resistance for mania, panic disorder, post-traumatic stress disorder (PTSD) and substance dependence. This is surprising given the evidence that nonresponse to standard therapies is a common clinical challenge in the treatment of these disorders [19–21], and represents a key outstanding issue for the field. It is not clear why this is the case but may reflect the fact that drug treatments for some of these disorders are less well established than for conditions like schizophrenia and major depression. Another consideration for definitions is whether functional impact is needed in addition to on-going symptoms. Only one criterion required a quantitative assessment of functioning to define treatment response [22].

Finally, a core component of the concept of treatment resistance is that the patient has taken the prescribed medication. Despite this, Table 1 shows that assessment of medication adherence is rarely included as a criterion for treatment resistance, and only one guideline recommended checking plasma drug levels to assess adherence. Thus, there is a risk that non-adherent patients may be considered as treatment resistant, which has implications for clinical trials of drugs for treatment resistance. Over a third of patients with schizophrenia identified as ‘treatment resistant’ show evidence of poor adherence [23], and poor adherence is reported in 10–60% of patients with depression [24]; this could mean that a large proportion of patients entering a trial are non-adherent rather than treatment resistant, potentially obscuring an effect or biasing results. Non-adherence is one contributor to pseudo-resistance, which is considered in the next section. Table 1 also shows that, where adherence is mentioned, most of the guidelines use statements such as ‘ensure adherence’ or ‘exclude poor adherence’ without making clear what constitutes adherence or poor adherence.

Overall, our review of the criteria shows considerable variability and lack of specificity in definitions of treatment resistance across psychiatric disorders, which has both clinical and research implications. The lack of clear definitions and, thus, risk of marked differences in patient characteristics between studies could be addressed by the use of standardised rating scales with established psychometric properties coupled with operationalising criteria to provide clear cut-offs. Heterogeneity could be reduced by harmonising criteria across guidelines within a disorder. Difficulties in knowing if studies recruited similar patients could be addressed by using a reporting checklist for treatment resistance that make clear, how each of the three components of treatment resistance was established in patients, an example of which is provided in Box 1.

Box 1 A treatment resistance reporting checklist for clinical trials and other studies to use in demonstrating on what basis patients with treatment resistant psychiatric disorders are recruited

1. **Correct diagnosis**
  - Was a standardised diagnostic tool used, and if so, what was it?
  - Was diagnostic stability confirmed, and if so, how was this assessed?
2. **Adequate treatment**
  - Was a prospective evaluation required?
  - What dose and duration of treatment was deemed to be an adequate trial?
  - How many adequate treatment trials were required?
  - Was adherence assessed, and if so how (e.g., use of drug plasma levels)?
3. **Non-response**
  - Was a prospective evaluation required?
  - How was response assessed? Specify the scales or tools used and time period.
  - If quantitatively assessed, what thresholds were used to distinguish adequate from inadequate response?
  - Was whether this is primary or secondary treatment resistance determined? If so, how?

### Primary versus secondary treatment resistance

Longitudinal studies indicate that trajectories to treatment resistance vary [25]. In some patients the illness shows an inadequate response to treatment from first presentation, whilst in others it initially shows a good response to treatment but over time this declines [25]. These prototypic trajectories have been respectively termed primary and secondary treatment resistance [26], although this terminology has been criticised as implying different mechanisms that are not known [22]. Alternatively, the descriptive terms ‘early onset’ and ‘late onset’ of treatment resistance may be used [22]. Notwithstanding discussions on terminology, this distinction is potentially important for research and clinical practice as it is unclear if treatments will be equally effective in these groups [22]. Putative neurobiological mechanisms for primary versus secondary treatment resistance are described below. Furthermore, a proportion of therapeutic benefit is derived from non-specific effects, which may be larger earlier in the course of illness [27].

The distinction between primary and secondary treatment resistance is only referred to in one of the consensus statements or guidelines recorded in Table 1 [22]. In this context, it would be helpful for future studies to investigate if there are differences between patients with primary versus secondary treatment resistance in treatment outcomes or neurobiology. Where this is not possible or warranted, clear reporting of the relative proportion of patients with primary versus secondary treatment resistance would help enable comparisons with other studies.

### Pseudo-resistance: diagnostic and treatment-related factors

Pseudo-resistance describes the circumstance where a patient’s condition does not respond to treatment, but the criteria for treatment resistance have not been fulfilled; for example, because the diagnosis was incorrect or the exposure to treatment was not adequate. Diagnostic instability, especially early in the course of a mental illness, is well described [28]. For example, bipolar affective disorder may initially present with a major depressive episode and be treated as unipolar depression. If such a patient has a poor outcome because of a treatment emergent mixed state or worsening of selected symptoms (e.g., insomnia or agitation), the patient may be deemed to have treatment resistant depression, when in actuality the wrong treatment had been selected [29]. This scenario highlights the need to ensure careful history and assessment of patients and cautions against concluding treatment resistance after only a short presentation.

**Fig. 3:**
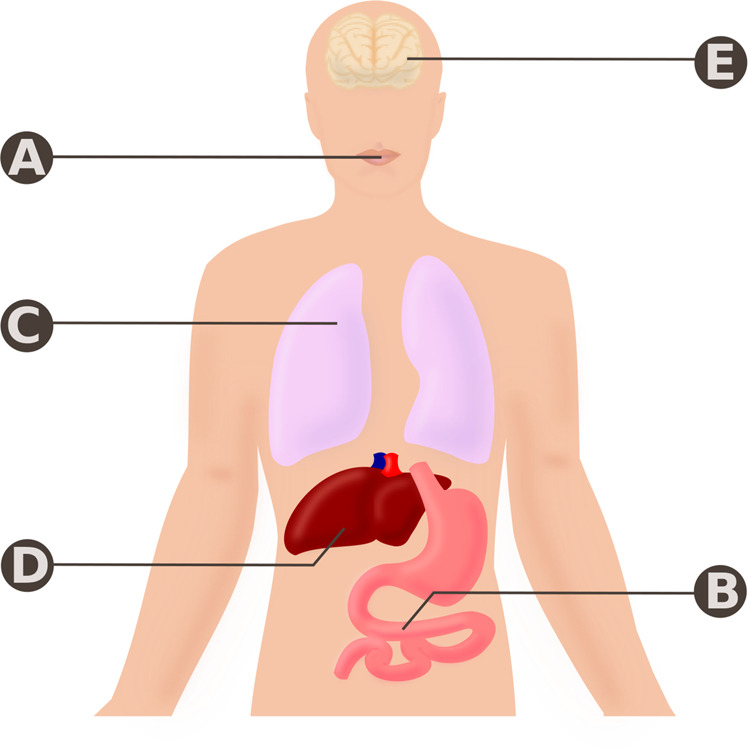
Pseudo-resistance to treatment in psychiatry: treatment related factors. **A** Poor concordance with medication or forgetfulness may result in insufficient drug being taken to achieve a therapeutic response, **B** Polymorphisms in P-glycoproteins in the gut endothelia may result in poor absorption of drugs and insufficient drug exposure. **C** Smoking tobacco induces expression of CYP450 enzymes, particularly CYP1A2, in the liver (**D**) resulting in enhanced break down of psychiatric medication metabolised by these enzymes. Polymorphisms in CYP450 enzymes that enhance their activity or co-administration of other psychiatric/non-psychiatric medications that act as enzyme inducers will have a similar effect. **E** Poor brain accumulation of drug owing to poor blood brain barrier permeability and/or polymorphisms in P-glycoprotein may result in insufficient central nervous system drug levels to achieve a therapeutic response.

Another common cause of pseudo-resistance is an inadequate therapeutic trial of treatment. Psychiatric drugs need to cross the blood-brain barrier and bind to their target in the brain. Several factors can lead to insufficient drug reaching the target in the brain. These are summarised in Table 2 and Fig. 3 (see eTable 1 for additional evidence). They include non-concordance with treatment, poor absorption of oral medication at the level of the gut endothelia, fast metabolism of medication by the liver, and poor blood-brain-barrier penetrance of medication. Some treatments may also be associated with a bell-shaped dose-response curve, where increasing dose leads to increasing efficacy only up to a point, whereupon further dose increases lead to decreasing efficacy [30]; in this scenario, high doses of treatment may be responsible for pseudo-resistance.

**Table 2:** Potential contributors to pseudo-resistance in schizophrenia and depression.

	Schizophrenia	Depression
Drug plasma levels and adherence	Over one third of patients identified as ‘treatment resistant’ show evidence of poor adherence [23]	A cross-sectional study observed that 15% of patients with MDD presenting with poor clinical response to tricyclic antidepressant therapy had ‘unusually low plasma concentrations relative to dose’ [119]. Poor adherence is reported in 10–60% of patients with depression [24]
Genetic variants affecting trans-membrane transporters	P-glycoprotein transporter polymorphisms influence antipsychotic response in schizophrenia [120]	P-glycoprotein transporter polymorphisms predict treatment response in depression [121]
Genetic variants affecting liver drug metabolism	Both first-generation and second-generation antipsychotics plasma levels and/or efficacy reduced by some CYP1A2, 2D6 and 3A4 polymorphisms [122]	Ultra-rapid metabolizer capacity recognised with polymorphisms of certain CYP450 enzymes (e.g., CYP2D6 and CYP2C19) result in reduced plasma levels for several antidepressants, including TCAs, SSRIs and SNRIs, and influence clinical response [123]
Liver drug metabolism: influence of co-prescribed psychiatric medication	Co-prescription of psychiatric medications that act as CYP450 inducers (e.g., lamotrigine and carbamazepine) can reduce plasma levels of some antipsychotics [124]	Co-prescription of psychiatric medications that act as CYP450 inducers (e.g., lamotrigine, carbamazepine) can reduce plasma levels of some antidepressants, including TCAs, SSRIs and bupropion [125]
Liver drug metabolism: influence of co-prescribed physical health medication	Co-prescription of medications that act as CYP450 inducers (e.g., omeprazole, phenytoin, St John’s wort, rifampicin) can reduce plasma levels of some antipsychotics [122]	Co-prescription of medications that act as CYP450 inducers (e.g., St John’s wort, phenytoin) may reduce plasma levels of some antidepressants [126]
Tobacco smoking	Smoking reduces plasma levels of those antipsychotics metabolised via CYP1A2 (e.g., olanzapine, clozapine) [127]	Smoking reduces plasma concentrations of various antidepressants [128]
Sex	Male gender predicts lower plasma levels of some antipsychotics [129]	Male gender predicts lower plasma levels of some antidepressants [130]
Alternative Diagnosis	Symptoms of other disorders, such as bipolar affective disorder, obsessive compulsive disorder or autism spectrum disorder, may be mistaken for schizophrenia [31, 32]	A minority of apparently resistant unipolar depression may in fact be depression associated with bipolar disorder [33, 34]

Further evidence is provided in eTable 1.

*TCA* tricyclic anti-depressant, *SSRI* selective serotonin reuptake inhibitor, *SNRI* serotonin and norepinephrine reuptake inhibitor.

### The clinical assessment of treatment resistance

**Fig. 4:**
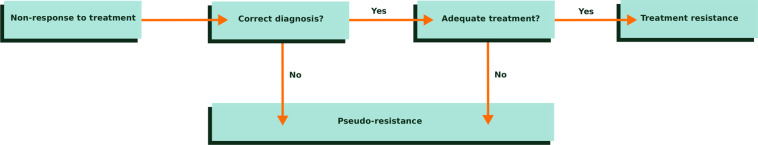
Algorithm for approaching non-response to treatment in psychiatric illness. Persistent symptoms despite treatment could be due to treatment resistance or due to other factors that give the impression of treatment resistance when in fact adequate treatment has not been received (pseudo-resistance). Pseudo-resistance may be secondary to an incorrect primary diagnosis/psychiatric comorbidity/substance abuse, or be treatment related, including poor treatment adherence, malabsorption of drug, poor blood brain barrier penetrance of drug or fast metabolism of drug (see Table 2 and Fig. 3).

The clinical assessment of potential treatment resistance is summarised in Fig. 4. A key step is to rule out pseudo-resistance as far as possible (Table 2 and Fig. 3). As such, clinical assessment should rule out alternate psychiatric diagnoses, e.g., features of autism spectrum disorder or OCD which may be mistaken for schizophrenia [31, 32] or bipolar-type depression which may be mistaken for unipolar depression [33, 34]. Where possible, duration and severity of symptoms, associated distress, and level of functioning should be quantified using validated clinical rating scales, which facilitates objective and accurate longitudinal assessment of treatment response. A comprehensive medication history should be taken to ensure that previous psychiatric medication trials were adequate, documenting dose and duration, and associated response. Finally, current treatment should be defined, and plasma levels measured where possible. This process should allow the assessment of key factors requisite for determining treatment resistance summarised in Fig. 4.

### Epidemiology and impact of treatment resistance

Prevalence estimates of TRS, TRD and treatment resistant OCD vary from 20 to 60% of the population with the condition [26, 35–40]. We were unable to identify prevalence data for treatment resistant bipolar affective disorder (whether depression or mania) or PTSD. Differences in the definitions of resistance between studies, as discussed above, are certain to substantially determine this variation. Another important factor is the population from which prevalence estimates are calculated, which can lead to ascertainment biases. For example, studies examining hospital populations with chronic illness are likely to record higher rates of treatment resistance compared with studies examining outpatient samples made of patients at the onset of illness.

In the USA, annual direct medical costs associated with TRS are conservatively estimated at over $34 billion [41]. It has been estimated that hospitalisation costs and total health resource utilisations for TRS are 10-fold higher than those for non-TRS, and that up to 80% of the total yearly health costs associated with schizophrenia in the USA are attributed to TRS [41]. Direct medical costs associated with TRD are estimated to be 2–6 times higher compared with other patients with MDD [42, 43], with costs increasing with chronicity and severity of TRD [44]. Patients with TRD are twice as likely to be hospitalised compared with non-TRD patients, and require increased numbers of psychiatric outpatient visits [43]. Patients with TRS have more functional impairment in the community compared with other serious mental illnesses [45], and the mean quality of life is estimated to be 20% lower in TRS compared with non-TRS [41]. Mean quality of life is approximately 25–40% lower in patients with TRD compared with patients who respond to treatment or who are in remission [46], and 30% of TRD patients attempt suicide in their lifetime [47].

### The neurobiology of treatment resistance

**Fig. 5:**
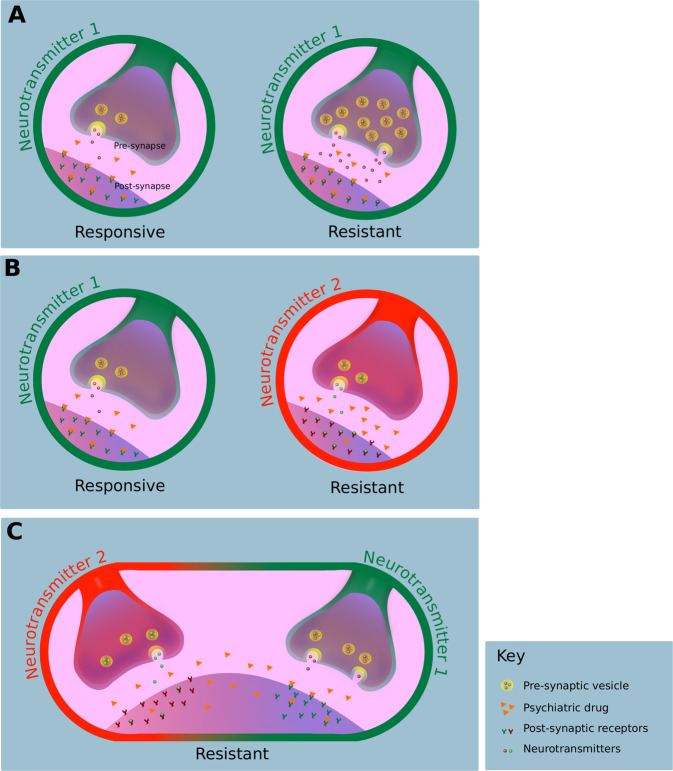
Main putative disease models to explain treatment resistance in psychiatry. **A** Treatment responsive and resistant illnesses are defined by the same neurobiological alterations (neurotransmitter 1), however the alterations are more marked in patients with treatment resistance. As such, higher doses of drugs targeting neurotransmitter system 1 (orange triangles) are required for therapeutic benefit. **B** Treatment responsive and resistant illnesses are defined by different underlying neurobiological mechanisms, for example two different neurotransmitter systems (neurotransmitters 1 and 2). As such, a drug targeting only neurotransmitter system 1 will be ineffective in patients with dysregulation in neurotransmitter system 2. **C** Treatment resistance arises from a combination of neurobiological alterations seen in responsive illness (neurotransmitter 1) in combination with a different neurobiological process (neurotransmitter 2). As such, treatments that act on both targets are likely to be needed.

There are three broad disease models proposed by the literature to explain treatment resistance, summarised in Fig. 5 (see eAppendix 3 for search details) [48–51]. The first possibility (Fig. 5A) is that the treatment resistant form of a given psychiatric illness has the same underlying neurobiology as the treatment responsive illness, but the pathophysiological alterations are more severe such that standard treatment is inadequate. This is partly supported by evidence that resistant symptoms of depression and schizophrenia may improve with higher doses of treatment in some patients [52–54]. Furthermore, as discussed above, secondary treatment resistance may be a consequence of either progressive neurobiological changes (pathoplasty) or iatrogenic effects. For example, in schizophrenia, an upregulation in dopamine D2/3 receptor levels with treatment could mean that a given antipsychotic dose is no longer enough to block dopaminergic neurotransmission adequately, leading to break-through resistant symptoms and necessitating higher doses [48].

Alternatively, treatment resistance could be neurobiologically distinct from treatment responsive illness (Fig. 5B). Supporting this, in some individuals, their illness shows little or no benefit from adequate treatment from illness onset despite evidence of high target engagement [55, 56], and there is some evidence of neurobiological differences between patients with TRS or TRD [57–59]. For example, studies using proton magnetic resonance spectroscopy have observed that levels of glutamatergic metabolites are particularly elevated in the anterior cingulate cortex in TRS compared to levels in patients, who respond to antipsychotic treatment and healthy controls and genetic and post-mortem studies also implicate glutamatergic pathways in TRS [60–62]. It should also be recognised that secondary treatment resistance may theoretically be a consequence of iatrogenic-change in neurotransmitter systems other than those primarily targeted by drugs, owing to off-target receptor actions.

The third possibility (Fig. 5C) is a hybrid model where patients show a combination of both the same pathophysiology as responsive illness and additional neurobiological alterations meaning that standard treatment is inadequate. One example is that of immune dysregulation. Converging lines of genetic, post-mortem, and pre-clinical evidence suggest immune dysregulation may play a role in the pathogenesis of both schizophrenia [63–67] and depression [68, 69], and it is hypothesised that immune alterations are seen only in a proportion of these patients (immune/inflammatory subgroups) and are linked to poor treatment response [67, 70–72]. For example, in depression, levels of C-reactive protein (CRP) are higher in TRD compared with those who respond to antidepressant treatment [73]. Furthermore, some [74], but not all [75], clinical trials examining immunotherapy in depression have observed efficacy only in patients with peripheral inflammation. Immune dysregulation has been implicated in dysregulation of multiple neurotransmitter systems [76–78] that some antipsychotic and antidepressant agents may not therapeutically target. Thus, without specifically targeting immune dysregulation alongside conventional therapy, symptoms will persist. This highlights the need to better understand the neurobiology of treatment resistance to help guide treatment design. Furthermore, it introduces the possibility that drugs previously deemed ineffective on a group-level may yet provide benefit to subgroups of patients.

### Therapeutic approaches to treatment resistance

For a drug to be considered in the management of treatment resistant psychiatric illness, its efficacy and specificity for use in treatment resistance must be demonstrated. Although studies that test drugs against placebo (or with no treatment) control for the scenario where symptoms improve with time, they do not control for the fact that longer treatment duration with an existing medication may lead to response. Thus, to demonstrate efficacy, the agent should demonstrate superiority over active comparators. Pharmacotherapy strategies for treatment resistance can be divided into two groups: monotherapies and adjunctive strategies.

To date, the only drug that has been licensed as monotherapy for a treatment resistant condition in psychiatry is clozapine for TRS, which gained regulatory approval on the basis of two studies [12, 79]. Although clozapine is a dopamine antagonist, relative to other antipsychotics it exhibits low D2 dopamine receptor occupancy [80], suggesting that disease model 1 is unlikely and modulation of other neurotransmitter systems may play a therapeutic role [81]. This is most in keeping with disease model 2 of treatment resistance described above (Fig. 5B), although it should be recognised that this needs testing and the mode of action of clozapine for TRS remains unclear.

Two adjunctive strategies for treatment resistance have been approved, both for TRD. Esketamine in combination with antidepressant therapy has recently received approval in both the US and Europe [82]. Esketamine blocks the NMDA receptor channel, and there is evidence that it induces plastic changes at glutamate synapses to improve connectivity [83], and may also affect other neurotransmitters [84]. The novel mechanism of action and efficacy of esketamine as an adjunct to conventional antidepressants (serotonin/norepinephrine reuptake inhibitors) are in keeping with disease model 3 (Fig. 5C). In the US, olanzapine combined with fluoxetine is a licensed therapy for TRD. FDA approval was based on data from studies that examined the olanzapine/fluoxetine combination compared with olanzapine or fluoxetine monotherapy [85–87]. These studies were 8-weeks in duration, and defined TRD as depression meeting DSM-IV criteria with persistence of symptoms despite at least two different antidepressant trials, including a prospective course of fluoxetine monotherapy. In all trials, depressive symptoms of patients who met criteria for treatment resistance improved significantly more when treated with the olanzapine/fluoxetine combination versus olanzapine or fluoxetine monotherapy. There is also evidence that adjunctive aripiprazole alongside antidepressant therapy is effective in people with MDD and inadequate response to antidepressant monotherapy [88]; furthermore, the magnitude of symptomatic improvement with adjunctive therapy was observed to be greater in those with only minimal response to antidepressant monotherapy compared to those with partial response [88]. The superior efficacy of combination therapy points towards patients with TRD presenting with a combination of the same neurochemical abnormality seen in first-line treatment responders with an additional neurochemical dysfunction, which is most in keeping with disease model 3 of treatment resistance detailed above (Fig. 5C).

As mentioned above, there is also some evidence that high doses of certain antidepressants (e.g., venlafaxine) [53, 89] or antipsychotics (e.g., olanzapine) [52] can be effective strategies in the management of treatment resistant depression and schizophrenia respectively. In the case of venlafaxine, there is evidence that increasing the dose beyond 150 mg/day results in a greater effect on noradrenergic neurotransmission [90]. This approach is most in keeping with disease model 1 of treatment resistance detailed above (Fig. 5A), although it should be recognised that, at higher doses, drug actions at other receptors may become more important.

In terms of neuro-stimulatory treatments, the FDA has approved use of transcranial magnetic stimulation in the treatment of MDD in patients who have failed to achieve ‘satisfactory improvement from one prior antidepressant medication’ [91]. However, it is unclear if such a definition reliably describes true treatment resistance (see ‘Current definitions of treatment resistance across disorders’ section above), and the FDA warns that efficacy of transcranial magnetic stimulation has not been established in patients with ‘varying degrees of medication resistance’. Electroconvulsive therapy (ECT) has widespread regulatory approval for treatment resistant depression and mania. The mechanism by which ECT effects response where medication has been unsuccessful remains poorly defined, but may stem from alterations in cerebral blood flow and regional metabolism [92]. This novel mechanism of action is in keeping with disease model 2 of treatment resistance detailed above (Fig. 5B).

### New treatment directions

To identify new treatments in the pipeline, we searched clinicaltrials.gov for phase 2 and 3 studies examining novel agents and interventions specifically for treatment resistant depression, schizophrenia, bipolar affective disorder, OCD, panic disorder, post-traumatic stress disorder, and substance dependence (see eAppendix 4 for search details). We identified 15 studies currently recruiting, covering treatments for schizophrenia, depression, bipolar affective disorder, and OCD. These are summarised in Table 3 (see eTable 2 for further details). They all have estimated completion dates within the next 2 years. Where stated, all the pharmacological interventions are adjunctive strategies, other than a clinical trial of LuAF35700 (an antagonist at dopaminergic, serotonergic and α-adrenergic receptors) as monotherapy for TRS that has just been completed. The headline results of this study indicate that it failed to demonstrate superiority over risperidone or olanzapine (clinical trials identifier: NCT02717195), although the full trial results remain to be published. In terms of non-pharmacological interventions, we identified studies examining transcranial magnetic stimulation in schizophrenia, vagus nerve stimulation in depression, deep brain stimulation in MDD and OCD, and radio-surgical (gamma capsulotomy) interventions in OCD.

**Table 3:** Novel interventions currently being examined in the management of treatment resistant schizophrenia, major depressive disorder, bipolar affective disorder, and obsessive compulsive disorder. Please see eTable 2 for further details and study links to clinicaltrials.gov.

Diagnosis and clinicaltrials.gov identifier	Compound or intervention	Mechanism of action	Mono/augmentation therapy	Study design	Estimated date of completion
Schizophrenia NCT03094429	Sodium benzoate	D-Amino acid oxidase inhibitor	Augmentation of clozapine	Placebo-control, phase 2/3	December 2021
Schizophrenia NCT03868839	Telmisartan	Angiotensin II receptor blocker	Augmentation	Single group, phase 2	October 2020
Schizophrenia NCT03762746	Transcranial magnetic stimulation	Targeting a putative source- monitoring deficit	Augmentation	Placebo-control, phase 3	February 2019 however still recruiting
Major depressive disorder NCT03775200	Psilocybin	Serotonin receptor agonist	Not stated	Single group, variable dose, phase 2	December 2020
Major depressive disorder NCT02660528	Tocilizumab	Anti-interleukin 6 receptor antibody	Not stated	Single group, open label, phase 2	November 2021
Major depressive disorder NCT03435744	Simvastatin	HMG-CoA reductase inhibitor	Augmentation	Placebo controlled, phase 3	December 2020
Major depressive disorder NCT03738215	Cariprazine	Dopamine D2/D3 receptor partial agonist	Augmentation	Placebo controlled, phase 3	July 2021
Major depressive disorder NCT03999918	Pimvanserin	Antagonist/inverse agonist at serotonin 5HT_2A_ receptors and less potently at 5HT_2C_ receptors	Augmentation	Placebo controlled, phase 3	August 2021
Major depressive disorder NCT04153812	Vagus nerve stimulation	Stimulation of vagal nerve afferent fibres	Not stated	Single group, open label, phase 2	May 2022
Major depressive disorder NCT04009928	Deep brain stimulation	Targeting the medial forebrain bundle or subcallosal cingulate	Not stated	Single group, cross-over (sham vs active), phase 2	January 2022
Bipolar affective disorder NCT03965871	Esketamine	NMDA-receptor antagonist	Augmentation	Placebo controlled, phase 2/3	April 2020
Obsessive compulsive disorder (OCD) NCT03184454	Deep brain stimulation	Targeting the dorsolateral prefrontal cortex and ventral anterior limb of internal capsule and adjacent ventral striatum	Unclear if monotherapy or augmentation	Single group, phase 2	October 2021
Obsessive compulsive disorder NCT04217408	Deep brain stimulation	Targeting the ventral capsule and ventral striatum	Unclear if monotherapy or augmentation	1-year of open-label treatment, followed by 5-week double blind on/off crossover, phase 2	May 2021
Obsessive compulsive disorder NCT03348930	Tolcapone	Catechol-O-methyltransferase inhibitor	Unclear if monotherapy or augmentation	Double blind placebo-controlled trial, phase 3	August 2020
Obsessive compulsive disorder NCT02433886	Bilateral single-shot ventral capsule/ventral striatum gamma capsulotomy	Radio-surgical induced lesion of brain region implicated in neurobiology of OCD	Unclear if monotherapy or augmentation	Single group, open label, phase 2	December 2020

### Outstanding issues and future directions

There are several outstanding issues in the field of psychiatric treatment resistance (Table 4). As discussed in the overview of operationalised descriptions of treatment resistance, there is marked heterogeneity in definitions used. This could lead to heterogeneity of study populations, which in turns limits the validity of research. Furthermore, for certain conditions such as mania, anxiety disorders, and PTSD, consensus definitions of resistance have yet to be agreed, and for some other conditions, such as autism spectrum disorder, there are no licenced drugs for core symptoms, thus, by definition, there is no treatment resistance. Although here we have predominantly focused on resistance to drug treatments, similar limitations apply to psychological and other therapies (e.g., in defining adequate treatment duration/number of psychology sessions). Furthermore, most approaches to treatment resistance consider the illness in terms of a single over-arching symptom dimension. However, these fail to recognise that some patients may have ongoing domain-specific disabling symptoms despite treatment, such as negative symptoms of schizophrenia or somatic symptoms of depression. Thus, the definitions of treatment resistance based on global rating may exclude people with one very disabling non-responsive symptom. Moreover, the imperfect nature of symptom rating scales may mean that degree of disability associated with some ratings of symptom severity are not captured. Finally, in the context of recognised phenomenological overlap between various psychiatric conditions, a transdiagnostic approach focussed on symptom domains, such as psychosis in schizophrenia and bipolar affective disorder, or depression in major depressive disorder and bipolar affective disorder [93], might help disentangle common underlying mechanisms linked to response and resistance. Clear objectives for the field are therefore to agree and adopt unified definitions of treatment resistance for each disorder, and to consider treatment resistance in terms of specific symptom domains (as already recommended by some definitions) [22] and their associated disability.

**Table 4:** Outstanding issues and future directions for research and clinical practice in the field of psychiatric treatment resistance.

Outstanding Issues	Proposed solutions and future directions
Marked heterogeneity in the definitions of treatment resistance provided by clinical guidelines, including in: • definitions of adequate treatment trials (e.g., drug dose and duration) • Number of treatment trials needed • Differentiation between non-response and partial response This will lead to the potential for inappropriate treatment decisions and bias in clinical trials	• Revision of definitions of treatment resistance to ensure greater specificity. Included in this effort should be recommendations to quantitatively assess treatment response and attempts to improve identification of pseudo-resistance e.g., through drug plasma monitoring to assess adherence • Harmonisation of definitions of treatment resistance across clinical guidelines • Clinical trials to cite criteria by which treatment resistance is defined and use reporting checklists for treatment resistance (see Box 1)
Absence of clear definitions of treatment resistance for various psychiatric conditions	• Definition of treatment resistance in conditions such as mania and post-traumatic stress disorder will facilitate appropriate clinical care and research
The neurobiology of treatment resistance in all psychiatric conditions remains unclear	• Efforts to determine the neural mechanisms underlying treatment resistance would help guide targeted and potentially personalised treatment approaches
The number of studies published annually on the topic of treatment resistance in psychiatry, although increasing, remains small (approximately 0.5% of papers published in the field of psychiatry in 2019). There is a paucity of treatments, particularly monotherapies, targeting treatment resistant symptoms in psychiatry	• Given that the prevalence of treatment resistance is up to 60% in some psychiatric populations, not to mention the significant associated economic and societal costs, more resources should be directed towards research in psychiatric treatment resistance and novel therapeutics. • Clinical trials in treatment resistance should aim to examine novel monotherapies versus an active comparator. • There is evidence that drugs already licenced for certain psychiatric disorders may have therapeutic potential in other treatment resistant psychiatric illnesses, e.g., clozapine for treatment resistant mania. Future clinical trials are required to establish an evidence base for their use in this context.

We identified circumstances that could lead to an illness being mistaken as treatment resistant, when non-response is in fact related to factors such as misdiagnosis or non-adherence (pseudo-resistance). Importantly, these factors are often not systematically assessed in research or clinical practice. Future work on identifying pseudo-resistance in the clinic and defining assessment pathways would be useful.

Greater understanding of the pathoaetiology of treatment resistance in psychiatric disorders could help identify new targets to guide the development of novel treatments. It could also help develop biomarkers to identify subgroups of patients with specific biological alterations that may benefit from tailored interventions earlier in the illness course, and to guide patient selection and evaluate target engagement in clinical trials. Furthermore, improved understanding of the mechanisms underlying different psychiatric symptom domains will provide insight into the transdiagnostic nature of their neurobiology and thus potential for overlapping treatments. Efforts to identify TRS based on genetics and neuroimaging are on-going [61, 94], and there has been considerable recent interest in stratifying patients with schizophrenia and depression based on blood immune markers with a view to identifying a subgroup whose psychiatric symptoms may respond to immunotherapy [67, 74, 75].

Strikingly, although the number of studies published annually on the topic of treatment resistance has increased over the last decade, the proportion of the literature dedicated to the topic of remains small (approximately 0.5% in 2019, see Fig. 1 and eAppendix 1). Given that the prevalence of treatment resistance in psychiatric conditions is 20–60%, and it is associated with high burden, there is a clear need for greater resources to be directed towards research in psychiatric treatment resistance.

## Conclusions

Treatment resistance comprises the trinity of establishing the correct psychiatric diagnosis, adequate treatment (in terms of dose and duration), and inadequate symptomatic response. It is commonly seen in many psychiatric disorders and associated with substantial functional impairment and economic and social costs. Whilst there has been a marked increase in research and industry interest in treatment resistance, the proportion of psychiatric research in this field remains very low relative to its burden. There is both marked variation and lack of clear criteria in the definition of treatment resistance both within and between disorders, which could lead to heterogeneity in the patients included in studies, making comparisons difficult. This highlights the need for greater consistency and operationalisation in the definitions of treatment resistance used, and we have provided a reporting checklist that future clinical trials can use to demonstrate on what basis patients with treatment resistance are recruited. There are only three pharmacological interventions licensed for treatment resistance, and only one, clozapine, licenced as a monotherapy. However, fifteen novel interventions are currently being examined in clinical trials of treatment resistant psychiatric conditions. Future drug development and clinical care will be informed by our improved understanding of the neurobiology of treatment resistance, and by employing various neuroimaging, molecular, and genetic techniques, may pave the way for precision medicine in the field.

## Supplementary information

Supplementary Information
