# Supplementary material for: Treatment resistance in psychiatry: state of the art and new directions
Source: Mol Psychiatry. 2021 Jul 13;27(1):58–72. doi: 10.1038/s41380-021-01200-3 (PMC8960394; doi:10.1038/s41380-021-01200-3)
Supplement: Supplementary file 1 — Supplementary Information [file 41380_2021_1200_MOESM1_ESM.docx]

**SUPPLEMENTARY INFORMATION**

**Treatment resistance in psychiatry: state of the art and new directions**

**Howes OD, Thase ME, Pillinger T**

**CONTENTS**

Pages 2-3 eAppendix 1: Search strategy to define rate of publication of articles focussing on treatment resistance in psychiatry and raw data

Page 4 eTable 1: Sources of pseudo treatment-resistance in schizophrenia and depression

Pages 5-6 eAppendix 2: Search strategy for definitions of treatment resistance in psychiatry

Page 6 eAppendix 3: Search strategy for reviews summarising current neurobiological understandings of treatment resistant schizophrenia and depression

Page 7 eAppendix 4: Search strategies for clinical trials of novel interventions in management of treatment resistant schizophrenia, depression, bipolar affective disorder, obsessive compulsive disorder, panic disorder, post-traumatic stress disorder, and substance dependence

Pages 8-9 eTable 2: Novel interventions currently being examined in the management of treatment resistant schizophrenia, depression, bipolar affective disorder, and obsessive compulsive disorder

Pages 10-13 Supplementary references

**eAppendix 1. Search strategy to define rate of publication of articles focussing on treatment resistance in psychiatry and raw data**

**Methods**

- Based on methods described in Zhang *et al*.^1^ we performed the following searches:
  - Web of Science was searched for total number of articles published annually in the field of psychiatry AND the topic of ‘treatment resistance’ between 2000-2019 inclusive. Search terms used: (WC=psychiatry AND TS=(‘treatment resistance’)); there were no language restrictions.
  - Web of Science was also searched for total number of articles published annually in the field of psychiatry overall between 2000-2019 inclusive. Search terms used: (WC=psychiatry); there were no language restrictions.
- For each year, the number for articles published in the field of psychiatry AND the topic ‘treatment resistance’ was divided by the number of articles published overall in the field of psychiatry, to give the ratio of publication on treatment resistance (TR) to the total number in the field (TR/total).
- We then performed a simple linear regression to examine the relationship between TR/total and year of publication. All analyses were performed in R (v3.5.1); the figure was produced using the ‘ggplot2’ package.

**Results**

- Raw data and annual TR/total ratios are presented below.
- There was a significant increase in the ratio of publications on TR to total publications in the field between 2000-2019: β=0.84, R^2^= 0.71, p<0.0001 (also see Figure 1 in main manuscript).

| Year | Papers published in field of treatment resistance in psychiatry | Total papers published in field of psychiatry | Ratio |
| --- | --- | --- | --- |
| 2000 | 39 | 16855 | 0.00231385 |
| 2001 | 53 | 14728 | 0.00359859 |
| 2002 | 48 | 18301 | 0.00262281 |
| 2003 | 74 | 18247 | 0.00405546 |
| 2004 | 71 | 23285 | 0.00304917 |
| 2005 | 64 | 22874 | 0.00279794 |
| 2006 | 73 | 22193 | 0.00328933 |
| 2007 | 85 | 22532 | 0.00377241 |
| 2008 | 99 | 24658 | 0.00401492 |
| 2009 | 105 | 23899 | 0.00439349 |
| 2010 | 102 | 25284 | 0.00403417 |
| 2011 | 105 | 27838 | 0.00377182 |
| 2012 | 119 | 29181 | 0.004078 |
| 2013 | 143 | 30933 | 0.0046229 |
| 2014 | 133 | 32208 | 0.00412941 |
| 2015 | 135 | 34458 | 0.00391781 |
| 2016 | 156 | 35690 | 0.00437097 |
| 2017 | 167 | 34740 | 0.00480714 |
| 2018 | 177 | 35553 | 0.00497848 |
| 2019 | 227 | 44208 | 0.00513482 |

**eTable 1. Sources of pseudo treatment-resistance in schizophrenia and depression**

|  | Schizophrenia | | Depression | |
| --- | --- | --- | --- | --- |
|  | **Representative Papers** | **Outcome** | **Representative Papers** | **Outcome** |
| Drug plasma levels and adherence | McCutcheon et al., 2018^2^ | Over one third of patients identified as ‘treatment resistant’ have subtherapeutic antipsychotic levels. Predictors of low plasma levels include black ethnicity and lower antipsychotic dose | Hollister et al., 1979^3^  Lingam et al., 2002^4^  Hunot et al., 2007^5^  Sawada et al., 2009^6^ | A cross-sectional study observed that 15% of patients with MDD presenting with poor clinical response to tricyclic antidepressant therapy had ‘unusually low plasma concentrations relative to dose’. Poor adherence is reported in 10-60% of patients with depression |
| Genetic variants affecting trans-membrane transporters | Nikisch et al., 2010^7^  Bozina et al., 2008^8^  Lin et al., 2006^9^  Kuzman et al., 2008^10^  Xing et al., 2006^11^ | P-glycoprotein (expressed on blood brain barrier and intestinal epithelia) polymorphisms may influence antipsychotic levels/pharmacokinetics/symptom severity in schizophrenia | Uhr et al., 2008^12^  Fukui et al., 2007^13^  Nikisch et al., 2008^14^  Gex-Fabry et al., 2008^15^  Sarginson et al., 2010^16^  O’Brien et al., 2012^17^  Kato et al., 2008^18^ | P-glycoprotein transporter polymorphisms predict treatment response in depression, and influence plasma levels/ pharmacokinetics of antidepressants |
| Genetic variants affecting liver drug metabolism | Perera et al., 2013^19^  Du et al., 2010^20^  Laika et al., 2010^21^  Llerena et al., 2004^22^  Someya et al., 2003^23^  Linnet et al., 1996^24^  Jerling et al., 1996^25^ | Rapid metabolism of psychiatric drugs owing to polymorphisms in hepatic cytochrome P450 enzymes leads to lower plasma levels of drugs. Both first- and second-generation antipsychotics plasma levels and/or efficacy reduced by some CYP1A2, 2D6, and 3A4 polymorphisms | Baumann et al., 1998^26^  Bertilsson et al., 1993^27^  Rau et al., 2004^28^  Fabbri et al., 2018^29^  Gex-Fabry et al., 2008^15^ | Ultra-rapid metabolizer capacity recognised with polymorphisms of certain CYP450 enzymes (e.g. CYP2D6 and CYP2C19) result in reduced plasma levels for several antidepressants, including TCAs, SSRIs, and SNRIs, and influence clinical response |
| Liver drug metabolism:  influence of co-prescribed psychiatric medication | Skogh et al., 2002^30^  Lucas et al., 1996^31^  Olesen et al., 1999^32^  Savasi et al., 2002^33^ | Co-prescription of psychiatric medications that act as CYP450 inducers (e.g. lamotrigine and carbamazepine) can reduce plasma levels of some antipsychotics | Leinonen et al., 1991^34^  Ketter et al., 1995^35^  De la Fuente et al., 1992^36^  Popli et al., 1995^37^ | Co-prescription of psychiatric medications that act as CYP450 inducers (e.g. lamotrigine, carbamazepine) can reduce plasma levels of some antidepressants, including TCAs, SSRIs, and bupropion |
| Liver drug metabolism:  influence of co-prescribed physical health medication | Savasi et al., 2002^33^  Frick et al., 2003^38^  Kennedy et al., 2013^39^  Van Strater et al., 2012^40^ | Co-prescription of physical health medications that act as CYP450 inducers (e.g. omeprazole, phenytoin, St John’s wort, rifampacin) can reduce plasma levels of some antipsychotics | Johne et al., 2002^41^  Perucca et al., 1985^42^ | Co-prescription of physical health medications that act as CYP450 inducers (e.g. St John’s wort, phenytoin) may reduce plasma levels of some antidepressants |
| Smoking | Patel et al., 2011^43^  Citrome et al., 2009^44^  Skogh et al., 2002^30^  Tsuda et al., 2014^45^ | Smoking reduces plasma levels of those antipsychotics metabolised via CYP1A2 (e.g. olanzapine, clozapine) | Oliveira et al., 2017^46^  Spigset et al., 1995^47^  Augustin et al., 2018^48^  Ishida et al., 1995^49^ | Smoking reduces plasma concentrations of various antidepressants |
| Sex | Patel et al., 2011^43^  Skogh et al., 2002^30^ | Male gender predicts lower plasma levels of some antipsychotics, potentially owing to higher CYP1A2 and CYP1A3 activity in males. | Bigos et al., 2009^50^  Kokras et al., 2011^51^ | Male gender predicts lower plasma levels of some antidepressants |
| Alternative Diagnosis | O’Dwyer et al., 2000^52^  King et al., 2011^53^ | Symptoms of psychosis may overlap with obsessive compulsive disorder or autism spectrum disorder. | Angst et al., 2003^54^  Smith et al., 2011^55^ | A minority of apparently resistant unipolar depression may in fact be bipolar-type depression. |

**eAppendix 2. Search strategy and results for definitions of treatment resistance in psychiatry**

We conducted a systematic review of national and international definitions of treatment resistance for common psychiatric diagnoses. We limited this to psychiatric disorders listed by the World Health Organisation in the 2013 global burden of disease study.^56^ As such, we systematically searched Pubmed for guidelines and consensus statements regarding diagnosis of the following disorders: schizophrenia, bipolar affective disorder (BPAD), Major Depressive Disorder (MDD), panic disorder, Obsessive Compulsive Disorder (OCD), Post-Traumatic Stress Disorder (PTSD), and alcohol/drug dependence. Specifically, the search was run as follows:

Pubmed was searched from inception to 25^th^ April 2019 using the following search terms: (treatment resistan*) AND (schizophrenia OR bipolar OR depression OR panic OR obsessive compulsive disorder OR post traumatic stress disorder OR dependence OR addiction). Selection criteria were: documents that contained definitions of treatment resistance in psychiatry (for schizophrenia, bipolar affective disorder, depression, panic disorder, Obsessive Compulsive Disorder, Post-Traumatic Stress Disorder, and alcohol/drug dependence) that were either consensus statements by groups of leading international researchers/clinicians in the field, or guidelines provided by established national/international Associations/Institutes (e.g. NICE, BAP).

This search was complemented by a separate search for management documents for schizophrenia, BPAD, anxiety disorders, OCD, PTSD, and addictions produced by nationally/internationally recognised psychiatric Associations/Institutes/Guidelines were examined for definitions of treatment resistance. Documents produced by the following bodies were examined: APA, RANZCP, MOHS, WFSBP, IPAP, NICE, BAP, Maudsley Guidelines.

American Psychiatric Association, Royal Australian and New Zealand College of Psychiatrists, Ministry of Health Singapore, World Federation of Societies of Biological Psychiatry, International Psychopharmacology Algorithm Project, National Institute for Health and Care Excellence, British Association for Psychopharmacology, and the Maudsley Guidelines.

The overarching search yielded 5887 records, of which 23 were included in the manuscript: 9 for schizophrenia, 10 for MDD, 1 for BPAD, and 3 for OCD. We were unable to identify definitions of treatment resistance for panic disorders, PTSD, or alcohol/other substance dependence. A PRISMA flowchart of the search process is shown below.

**
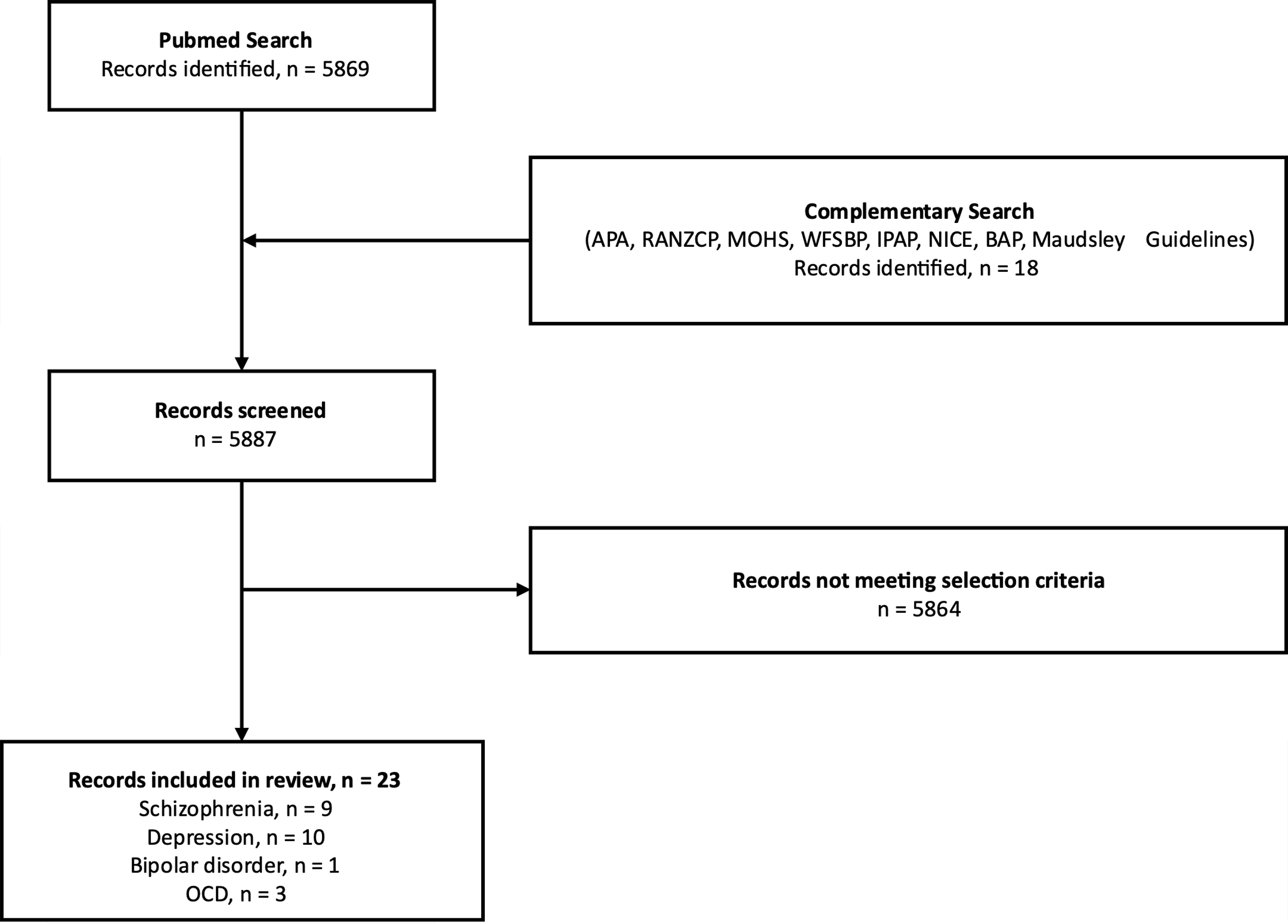
**

**eAppendix 3. Search strategy for reviews summarising current neurobiological understandings of treatment resistant schizophrenia and depression**

Pubmed was searched from inception to 25^th^ April 2019 using the following search terms: *(treatment resistan*) AND (schizophrenia OR depression),* filtering for reviews. Selection criteria were: review articles written in English that set out to provide summary evidence of putative mechanisms of treatment resistant schizophrenia or depression. A PRISMA flowchart of the search process is shown below.


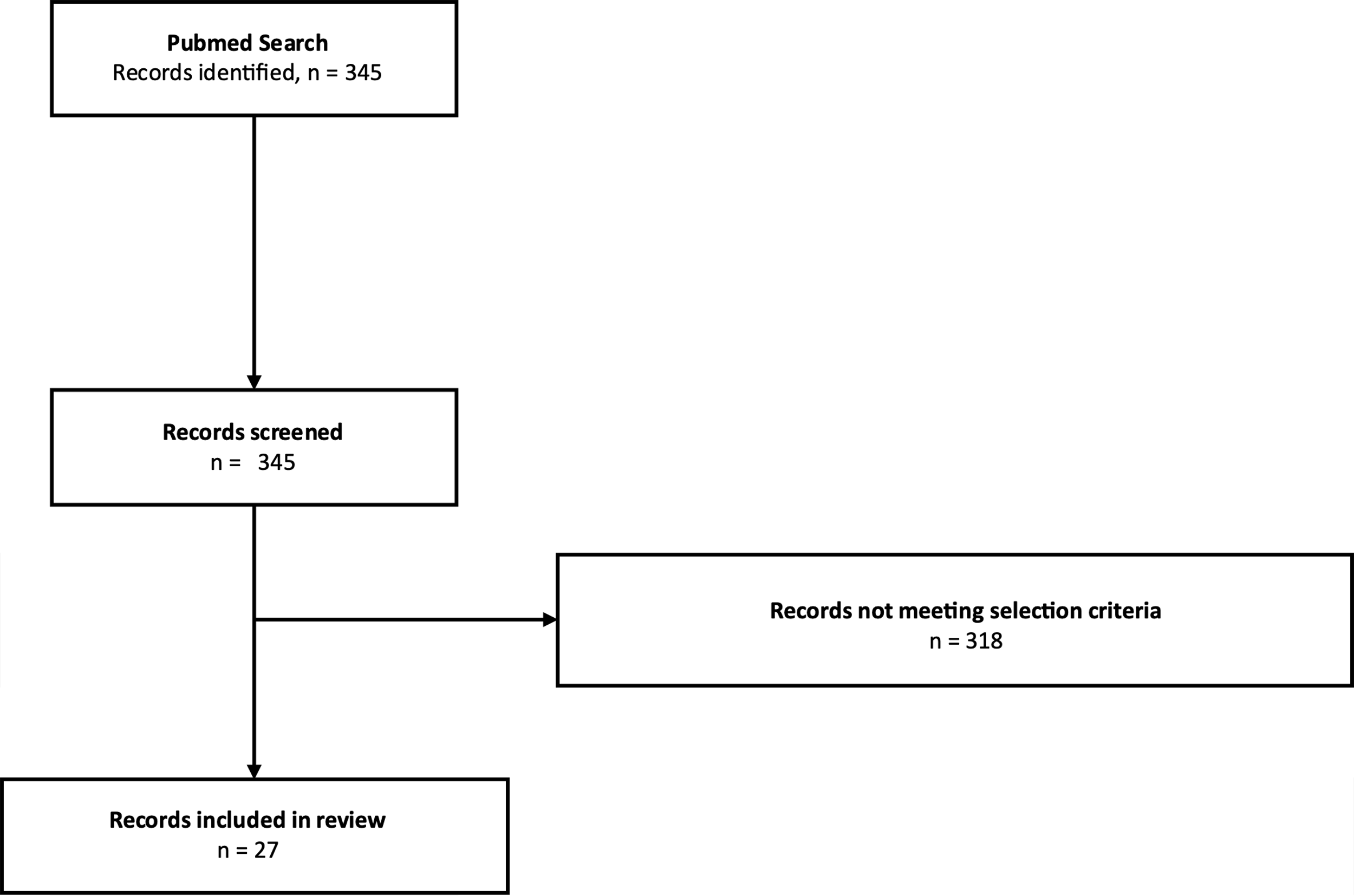


**eAppendix 4. Search strategies for clinical trials of novel interventions in management of treatment resistant schizophrenia, depression, bipolar affective disorder, obsessive compulsive disorder, panic disorder, post-traumatic stress disorder, and substance dependence**

Clinicaltrials.gov was searched from inception to 18^th^ March 2020 using the following search terms:

Condition: ‘schizophrenia’ or ‘depression’ or ‘bipolar affective disorder’ or ‘obsessive compulsive disorder’ or ‘panic’ or ‘post traumatic stress disorder’ or ‘dependence’ or ‘addiction’

Other terms: none

Country: any

Filters: Phase 2 and 3 trials, adults

Selection criteria: studies currently recruiting that are examining interventions in patients with treatment resistant schizophrenia, depression, bipolar affective disorder, obsessive compulsive disorder, panic disorder, post-traumatic stress disorder, and substance dependence; outcome measure: clinical rating scales. A PRISMA flowchart of the search process is shown below.

**
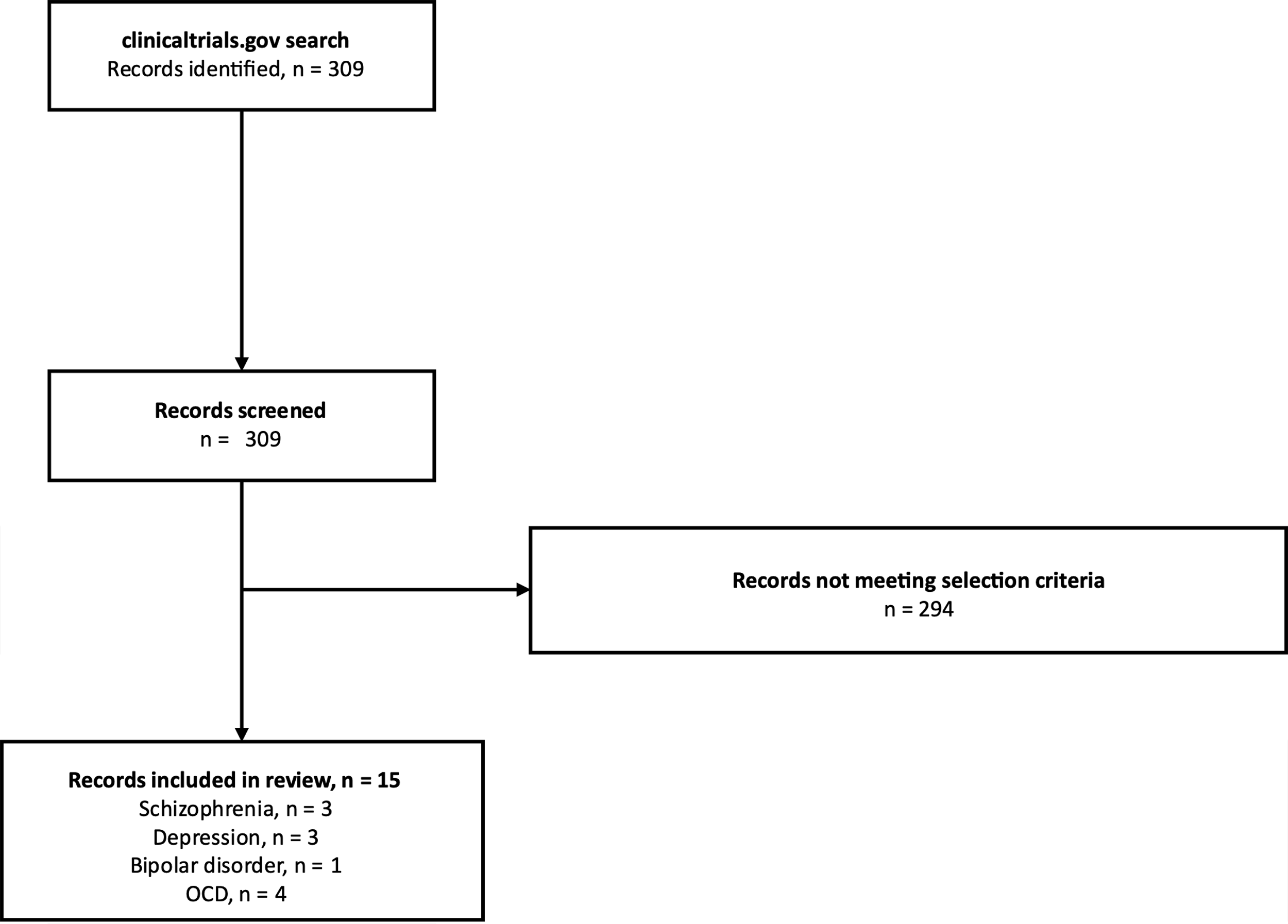
**

**eTable 2. Novel interventions currently being examined in the management of treatment resistant schizophrenia, depression, bipolar affective disorder, and obsessive compulsive disorder**

| Drug | Clinicaltrials.gov ID | Mechanism | Link and note |
| --- | --- | --- | --- |
| Schizophrenia (3 studies examined treatment resistance out of a total of 62 hits) | | | |
| Sodium benzoate (+TAU: add on to clozapine) | NCT03094429 | D-Amino Acid Oxidase Inhibitor | <https://clinicaltrials.gov/ct2/show/NCT03094429?recrs=a&cond=Schizophrenia&age=1&phase=12&draw=2&rank=5>  Placebo-control, phase 2/3  Estimated study completion date: December 2021 |
| Telmisartan (+TAU) | NCT03868839 | Angiotensin II receptor blocker | <https://clinicaltrials.gov/ct2/show/NCT03868839?recrs=a&cond=Schizophrenia&age=1&phase=12&draw=2&rank=32>  Single group, phase 2 (note symptom change not primary outcome)  Estimated study completion date: October 2020 |
| Transcranial magnetic stimulation (+TAU) | NCT03762746 | Targeting a putative source- monitoring deficit | <https://clinicaltrials.gov/ct2/show/NCT03762746?recrs=a&cond=Schizophrenia&age=1&phase=12&draw=2&rank=54>  Placebo-control. Phase 3  Estimated study completion date: February 2019 (however reported still to be recruiting) |
| Depression (7 studies examined treatment resistance out of a total of 115 hits) | | | |
| Psilocybin (+TAU assumed but not stated) | NCT03775200 | Serotonin receptor agonist | <https://clinicaltrials.gov/ct2/show/NCT03775200?recrs=a&cond=Depression&age=1&phase=12&draw=2&rank=3>  Single group, variable dose. Phase 2  Estimated study completion date: December 2020 |
| Tocilizumab (+TAU) | NCT02660528 | Anti-interleukin 6 receptor antibody | <https://clinicaltrials.gov/ct2/show/NCT02660528?recrs=a&cond=Depression&age=1&phase=12&draw=2&rank=17>  Single group, open label. Phase 2  Estimated study completion date: November 2021 |
| Simvastatin augmentation (+TAU) | NCT03435744 | HMG-CoA reductase inhibitor | <https://clinicaltrials.gov/ct2/show/NCT03435744?recrs=a&cond=Depression&age=1&phase=12&draw=2&rank=33>  Phase 3, placebo controlled  Estimated study completion date: December 2020 |
| Cariprazine (+TAU) | NCT03738215 | Dopamine D2/D3 receptor partial agonist | <https://clinicaltrials.gov/ct2/show/NCT03738215?recrs=a&cond=Depression&age=1&phase=12&draw=2&rank=64>  Phase 3, placebo controlled  Estimated study completion date: July 2021 |
| Pimvanserin (+TAU) | NCT03999918 | Antagonist/inverse agonist at serotonin 5HT2A receptors and less potently at 5HT2C receptors | <https://clinicaltrials.gov/ct2/show/NCT03999918?recrs=a&cond=Depression&age=1&phase=12&draw=2&rank=71>  Phase 3, placebo controlled  Estimated study completion date: August 2021 |
| Vagus nerve stimulation (assumed +TAU but not stated) | NCT04153812 | Stimulation of vagal nerve afferent fibres | <https://clinicaltrials.gov/ct2/show/NCT04153812?recrs=a&cond=Depression&age=1&phase=12&draw=2&rank=77>  Single group, open label. Phase 2  Estimated study completion date: May 2022 |
| Deep brain stimulation  (assumed +TAU but not stated) | NCT04009928 | Targeting the medial forebrain bundle or subcallosal cingulate (neural circuitry involved in panic and reward that may be altered in depression) | <https://clinicaltrials.gov/ct2/show/NCT04009928?recrs=a&cond=Depression&age=1&phase=12&draw=2&rank=78>  Single group, cross-over (sham vs active). Phase 2  Estimated study completion date: January 2022 |
| Bipolar Affective Disorder (1 study examined treatment resistance out of 32 hits) | | | |
| Esketamine +TAU | NCT03965871 | NMDA-receptor antagonist | <https://clinicaltrials.gov/ct2/show/NCT03965871?recrs=a&cond=Bipolar+Affective+Disorder&age=1&phase=12&draw=2&rank=22>  Phase 2/3 placebo-controlled  Estimated study completion date: April 2020 |
| Obsessive Compulsive Disorder (4 studies examined treatment resistance out of 6 hits) | | | |
| Deep Brain Stimulation (whether +TAU not stated) | NCT03184454 | Targeting the dorsolateral prefrontal cortex (dlPFC) and the ventral anterior limb of the internal capsule and adjacent ventral striatum (VC/VS); brain circuitry and associated functional dysconnectivity implicated in OCD | <https://clinicaltrials.gov/ct2/show/NCT03184454?cond=Obsessive-Compulsive+Disorder&age=1&phase=12&draw=2&rank=2>  Single group. Phase 2  Estimated study completion date: October 2021 |
| Deep Brain Stimulation (DBS)  (whether +TAU not stated) | NCT04217408 | Targeting the Ventral Capsule/Ventral Striatum (VC/VS); brain circuitry and associated functional dysconnectivity implicated in OCD | <https://clinicaltrials.gov/ct2/show/NCT04217408?cond=Obsessive-Compulsive+Disorder&age=1&phase=12&draw=3&rank=23>  Phase 2. 1 year of open-label treatment, followed by 5 week double blinded crossover (on/off) phase  Estimated study completion date: May 2021 |
| Tolcapone  +TAU (but not if psychology started within 3-months of study initiation) | NCT03348930 | COMT inhibitor | <https://clinicaltrials.gov/ct2/show/NCT03348930?cond=Obsessive-Compulsive+Disorder&age=1&phase=12&draw=3&rank=18>  Double blind RCT. Phase 3  Estimated study completion date: August 2020 |
| Bilateral single-shot ventral capsule/ventral striatum gamma capsulotomy  (whether +TAU not stated) | NCT02433886 | Radiosurgical induced lesion of brain region implicated in neurobiology of OCD | <https://clinicaltrials.gov/ct2/show/NCT02433886?cond=Obsessive-Compulsive+Disorder&age=1&phase=12&draw=3&rank=51>  Phase 2. Single group, open label  Estimated study completion date: December 2020 |

**Supplementary References**

1. Zhang J, Chen X, Gao X, et al. Worldwide research productivity in the field of psychiatry. *Int J Ment Health Syst.* 2017;11:20.

2. McCutcheon R, Beck K, Bloomfield MAP, Marques TR, Rogdaki M, Howes OD. Treatment resistant or resistant to treatment? Antipsychotic plasma levels in patients with poorly controlled psychotic symptoms. *Journal of Psychopharmacology.* 2015;29(8):892-897.

3. Hollister LE. Monitoring tricyclic antidepressant plasma concentrations. *JAMA.* 1979;241(23):2530-2533.

4. Lingam R, Scott J. Treatment non-adherence in affective disorders. *Acta Psychiatr Scand.* 2002;105(3):164-172.

5. Hunot VM, Horne R, Leese MN, Churchill RC. A cohort study of adherence to antidepressants in primary care: the influence of antidepressant concerns and treatment preferences. *Prim Care Companion J Clin Psychiatry.* 2007;9(2):91-99.

6. Sawada N, Uchida H, Suzuki T, et al. Persistence and compliance to antidepressant treatment in patients with depression: a chart review. *BMC Psychiatry.* 2009;9:38.

7. Nikisch G, Baumann P, Oneda B, et al. Cytochrome P450 and ABCB1 genetics: association with quetiapine and norquetiapine plasma and cerebrospinal fluid concentrations and with clinical response in patients suffering from schizophrenia. A pilot study. *J Psychopharmacol.* 2011;25(7):896-907.

8. Bozina N, Kuzman MR, Medved V, Jovanovic N, Sertic J, Hotujac L. Associations between MDR1 gene polymorphisms and schizophrenia and therapeutic response to olanzapine in female schizophrenic patients. *Journal of Psychiatric Research.* 2008;42(2):89-97.

9. Lin YC, Ellingrod VL, Bishop JR, Miller DD. The relationship between P-glycoprotein (PGP) polymorphisms and response to olanzapine treatment in schizophrenia. *Therapeutic Drug Monitoring.* 2006;28(5):668-672.

10. Kuzman MR, Medved V, Bozina N, Hotujac L, Sain I, Bilusic H. The influence of 5-HT2C and MDR1 genetic polymorphisms on antipsychotic-induced weight gain in female schizophrenic patients. *Psychiatry Research.* 2008;160(3):308-315.

11. Xing QH, Gao R, Li HF, et al. Polymorphisms of the ABCB1 gene are associated with the therapeutic response to risperidone in Chinese schizophrenia patients. *Pharmacogenomics.* 2006;7(7):987-993.

12. Uhr M, Tontsch A, Namendorf C, et al. Polymorphisms in the drug transporter gene ABCB1 predict antidepressant treatment response in depression. *Neuron.* 2008;57(2):203-209.

13. Fukui N, Suzuki Y, Sawamura K, et al. Dose-dependent effects of the 3435 C > T genotype of ABCB1 gene on the steady-state plasma concentration of fluvoxamine in psychiatric patients. *Therapeutic Drug Monitoring.* 2007;29(2):185-189.

14. Nikisch G, Eap CB, Baumann P. Citalopram enantiomers in plasma and cerebrospinal fluid of ABCB1 genotyped depressive patients and clinical response: A pilot study. *Pharmacological Research.* 2008;58(5-6):344-347.

15. Gex-Fabry M, Eap CB, Oneda B, et al. CYP2D6 and ABCB1 genetic variability: Influence on paroxetine plasma level and therapeutic response. *Therapeutic Drug Monitoring.* 2008;30(4):474-482.

16. Sarginson JE, Lazzeroni LC, Ryan HS, Ershoff BD, Schatzberg AF, Murphy GM. ABCB1 ( MDR1) polymorphisms and antidepressant response in geriatric depression. *Pharmacogenetics and Genomics.* 2010;20(8):467-475.

17. O'Brien FE, Dinan TG, Griffin BT, Cryan JF. Interactions between antidepressants and P-glycoprotein at the blood-brain barrier: clinical significance of in vitro and in vivo findings. *Brit J Pharmacol.* 2012;165(2):289-312.

18. Kato M, Fukuda T, Serretti A, et al. ABCB1 (MDR1) gene polymorphisms are associated with the clinical response to paroxetine in patients with major depressive disorder. *Progress in Neuro-Psychopharmacology & Biological Psychiatry.* 2008;32(2):398-404.

19. Perera V, Gross AS, Polasek TM, et al. Considering CYP1A2 phenotype and genotype for optimizing the dose of olanzapine in the management of schizophrenia. *Expert Opin Drug Met.* 2013;9(9):1115-1137.

20. Du J, Zhang A, Wang L, et al. Relationship between response to risperidone, plasma concentrations of risperidone and CYP3A4 polymorphisms in schizophrenia patients. *Journal of Psychopharmacology.* 2010;24(7):1115-1120.

21. Laika B, Leucht S, Heres S, Schneider H, Steimer W. Pharmacogenetics and olanzapine treatment: CYP1A2*1F and serotonergic polymorphisms influence therapeutic outcome. *Pharmacogenomics J.* 2010;10(1):20-29.

22. Llerena A, de la Rubia AC, Berecz R, Dorado P. Relationship between haloperidol plasma concentration, Debrisoquine metabolic ratio, CYP2D6 and CYP2C9 genotypes in psychiatric patients. *Pharmacopsychiatry.* 2004;37(2):69-73.

23. Someya T, Shimoda K, Suzuki Y, et al. Effect of CYP2D6 genotypes on the metabolism of haloperidol in a Japanese psychiatric population. *Neuropsychopharmacology.* 2003;28(8):1501-1505.

24. Linnet K, Wiborg O. Steady-state serum concentrations of the neuroleptic perphenazine in relation to CYP2D6 genetic polymorphism. *Clinical Pharmacology & Therapeutics.* 1996;60(1):41-47.

25. Jerling M, Dahl ML, AbergWistedt A, et al. The CYP2D6 genotype predicts the oral clearance of the neuroleptic agents perphenazine and zuclopenthixol. *Clinical Pharmacology & Therapeutics.* 1996;59(4):423-428.

26. Baumann P, Broly F, Kosel M, Eap CB. Ultrarapid metabolism of clomipramine in a therapy-resistant depressive patient, as confirmed by CYP2 D6 genotyping. *Pharmacopsychiatry.* 1998;31(2):72-72.

27. Bertilsson L, Dahl ML, Sjoqvist F, et al. Molecular-Basis for Rational Megaprescribing in Ultrarapid Hydroxylators of Debrisoquine. *Lancet.* 1993;341(8836):63-63.

28. Rau T, Wohlleben G, Wuttke H, et al. CYP2D6 genotype: Impact on adverse effects and nonresponse during treatment with antidepressants - A pilot study. *Clinical Pharmacology & Therapeutics.* 2004;75(5):386-393.

29. Fabbri C, Tansey KE, Perlis RH, et al. Effect of cytochrome CYP2C19 metabolizing activity on antidepressant response and side effects: Meta-analysis of data from genome-wide association studies. *European Neuropsychopharmacology.* 2018;28(8):945-954.

30. Skogh E, Reis M, Dahl ML, Lundmark J, Bengtsson F. Therapeutic drug monitoring data on olanzapine and its N-demethyl metabolite in the naturalistic clinical setting. *Ther Drug Monit.* 2002;24(4):518-526.

31. Lucas RA, Gilfillan DJ, Bergstrom RF. A pharmacokinetic interaction between carbamazepine and olanzapine: observations on possible mechanism. *European Journal of Clinical Pharmacology.* 1998;54(8):639-643.

32. Olesen OV, Linnet K. Olanzapine serum concentrations in psychiatric patients given standard doses: The influence of comedication. *Therapeutic Drug Monitoring.* 1999;21(1):87-90.

33. Savasi I, Millson RC, Owen JA. Quetiapine blood level variability. *Can J Psychiat.* 2002;47(1):94-94.

34. Leinonen E, Lillsunde P, Laukkanen V, Ylitalo P. Effects of Carbamazepine on Serum Antidepressant Concentrations in Psychiatric-Patients. *Journal of Clinical Psychopharmacology.* 1991;11(5):313-318.

35. Ketter TA, Jenkins JB, Schroeder DH, et al. Carbamazepine but Not Valproate Induces Bupropion Metabolism. *Journal of Clinical Psychopharmacology.* 1995;15(5):327-333.

36. Delafuente JM, Mendlewicz J. Carbamazepine Addition in Tricyclic Antidepressant-Resistant Unipolar Depression. *Biological Psychiatry.* 1992;32(4):369-374.

37. Popli AP, Tanquary J, Lamparella V, Masand PS. Bupropion and anticonvulsant drug interactions. *Ann Clin Psychiatry.* 1995;7(2):99-101.

38. Frick A, Kopitz J, Bergemann N. Omeprazole reduces clozapine plasma concentrations - A case report. *Pharmacopsychiatry.* 2003;36(3):121-123.

39. Kennedy WK, Jann MW, Kutscher EC. Clinically Significant Drug Interactions with Atypical Antipsychotics. *Cns Drugs.* 2013;27(12):1021-1048.

40. Van Strater ACP, Bogers JPAM. Interaction of St John's wort (Hypericum perforatum) with clozapine. *International Clinical Psychopharmacology.* 2012;27(2):121-123.

41. Johne A, Schmider J, Brockmoller J, et al. Decreased plasma levels of amitriptyline and its metabolites on comedication with an extract from St. John's Wort (Hypericum perforatum). *Journal of Clinical Psychopharmacology.* 2002;22(1):46-54.

42. Perucca E. Pharmacokinetic interactions with antiepileptic drugs. *Clin Pharmacokinet.* 1982;7(1):57-84.

43. Patel MX, Bowskill S, Couchman L, et al. Plasma olanzapine in relation to prescribed dose and other factors: data from a therapeutic drug monitoring service, 1999-2009. *J Clin Psychopharmacol.* 2011;31(4):411-417.

44. Citrome L, Stauffer VL, Chen L, et al. Olanzapine plasma concentrations after treatment with 10, 20, and 40 mg/d in patients with schizophrenia: An analysis of correlations with efficacy, weight gain, and prolactin concentration. *Journal of Clinical Psychopharmacology.* 2009;29(3):278-283.

45. Tsuda Y, Saruwatari J, Yasui-Furukori N. Meta-analysis: the effects of smoking on the disposition of two commonly used antipsychotic agents, olanzapine and clozapine. *BMJ Open.* 2014;4(3):e004216.

46. Oliveira P, Ribeiro J, Donato H, Madeira N. Smoking and antidepressants pharmacokinetics: a systematic review. *Ann Gen Psychiatry.* 2017;16:17.

47. Spigset O, Carleborg L, Hedenmalm K, Dahlqvist R. Effect of cigarette smoking on fluvoxamine pharmacokinetics in humans. *Clin Pharmacol Ther.* 1995;58(4):399-403.

48. Augustin M, Schoretsanitis G, Hiemke C, Grunder G, Haen E, Paulzen M. Differences in Duloxetine Dosing Strategies in Smoking and Nonsmoking Patients: Therapeutic Drug Monitoring Uncovers the Impact on Drug Metabolism. *J Clin Psychiatry.* 2018;79(5).

49. Ishida M, Otani K, Kaneko S, et al. Effects of Various Factors on Steady-State Plasma-Concentrations of Trazodone and Its Active Metabolite M-Chlorophenylpiperazine. *International Clinical Psychopharmacology.* 1995;10(3):143-146.

50. Bigos KL, Pollock BG, Stankevich BA, Bies RR. Sex differences in the pharmacokinetics and pharmacodynamics of antidepressants: an updated review. *Gend Med.* 2009;6(4):522-543.

51. Kokras N, Dalla C, Papadopoulou-Daifoti Z. Sex differences in pharmacokinetics of antidepressants. *Expert Opin Drug Met.* 2011;7(2):213-226.

52. O'Dwyer AM, Marks I. Obsessive-compulsive disorder and delusions revisited. *British Journal of Psychiatry.* 2000;176:281-284.

53. King BH, Lord C. Is schizophrenia on the autism spectrum? *Brain Res.* 2011;1380:34-41.

54. Angst J, Gamma A, Benazzi F, Ajdacic V, Eich D, Rossler W. Toward a re-definition of subthreshold bipolarity: epidemiology and proposed criteria for bipolar-II, minor bipolar disorders and hypomania. *Journal of Affective Disorders.* 2003;73(1-2):133-146.

55. Smith DJ, Griffiths E, Kelly M, Hood K, Craddock N, Simpson SA. Unrecognised bipolar disorder in primary care patients with depression. *British Journal of Psychiatry.* 2011;199(1):49-56.

56. Global Burden of Disease Study C. Global, regional, and national incidence, prevalence, and years lived with disability for 301 acute and chronic diseases and injuries in 188 countries, 1990-2013: a systematic analysis for the Global Burden of Disease Study 2013. *Lancet.* 2015;386(9995):743-800.
